# Relevance of *ARID1A* Mutations in Endometrial Carcinomas

**DOI:** 10.3390/diagnostics12030592

**Published:** 2022-02-25

**Authors:** Antonio De Leo, Gloria Ravegnini, Francesco Musiani, Thais Maloberti, Michela Visani, Viviana Sanza, Sabrina Angelini, Anna Myriam Perrone, Pierandrea De Iaco, Angelo Gianluca Corradini, Francesca Rosini, Marco Grillini, Donatella Santini, Claudio Ceccarelli, Claudio Zamagni, Giovanni Tallini, Dario de Biase

**Affiliations:** 1 Department of Experimental, Diagnostic and Specialty Medicine, University of Bologna-Molecular Diagnostic Unit, Azienda USL di Bologna, 40138 Bologna, Italy; antonio.deleo@unibo.it (A.D.L.); thais.maloberti2@unibo.it (T.M.); michela.visani@unibo.it (M.V.); giovanni.tallini@ausl.bologna.it (G.T.); 2 Division of Molecular Pathology, IRCCS Azienda Ospedaliero-Universitaria di Bologna, 40138 Bologna, Italy; viviana.sanza@ausl.bologna.it; 3 Department of Pharmacy and Biotechnology (FaBiT), University of Bologna, 40127 Bologna, Italy; gloria.ravegnini2@unibo.it (G.R.); s.angelini@unibo.it (S.A.); 4 Laboratory of Bioinorganic Chemistry, Department of Pharmacy and Biotechnology, University of Bologna, 40127 Bologna, Italy; francesco.musiani@unibo.it; 5 Division of Gynecologic Oncology, IRCCS Azienda Ospedaliero-Universitaria di Bologna, 40138 Bologna, Italy; myriam.perrone@aosp.bo.it (A.M.P.); pierandrea.deiaco@unibo.it (P.D.I.); 6 Department of Medical and Surgical Sciences (DIMEC)-Centro di Studio e Ricerca delle Neoplasie Ginecologiche (CSR), University of Bologna, 40138 Bologna, Italy; 7 Pathology Unit, IRCCS Azienda Ospedaliero-Universitaria di Bologna, 40138 Bologna, Italy; angelo.corradini@aosp.bo.it (A.G.C.); francesca.rosini@aosp.bo.it (F.R.); marco.grillini@aosp.bo.it (M.G.); donatella.santini@aosp.bo.it (D.S.); 8 Department of Experimental, Diagnostic and Specialty Medicine (DIMES), University of Bologna, 40138 Bologna, Italy; claudio.ceccarelli@unibo.it; 9 Addarii Medical Oncology, IRCCS Azienda Ospedaliero-Universitaria di Bologna, 40138 Bologna, Italy; claudio.zamagni@aosp.bo.it; 10 Department of Pharmacy and Biotechnology (FaBiT), University of Bologna-Molecular Diagnostic Unit, 40138 Bologna, Italy

**Keywords:** endometrial cancer, ARID1A, SWI/SNF complex, molecular classification, next-generation sequencing

## Abstract

Since the Cancer Genome Atlas (TCGA) project identified four distinct groups based on molecular alterations, mutation analyses have been integrated into the characterization of endometrial carcinomas (ECs). ARID1A seems to be the subunit more involved in the loss of function of the SWI/SNF complex in ECs. The aim of this study is to define the relevance of *ARID1A* alterations in a cohort of EC, studying the possible associations between DNA mutation (genomic level), RNA expression (transcriptomic level), and protein expression (proteomic level). A total of 50 endometrial carcinomas were characterized for *ARID1A* mutations (using targeted DNA next-generation sequencing—NGS), *ARID1A* gene expression (using RNAseq and qRT-PCR), and ARID1A protein expression (using immunohistochemistry—IHC). Moreover, we have investigated if *ARID1A* mutations may alter the protein structure, using the Protein Data Bank sequence. We found a good correlation between *ARID1A* mutations and protein immunostaining, even if we did not find statistically significant differences in the *ARID1A* expression levels. In conclusion, our data demonstrated that the molecular characterization of *ARID1A* should be associated with IHC analysis, mainly in those cases harboring “novel” *ARID1A* mutations or in those alterations with “uncertain” pathogenic significance.

## 1. Introduction

The annual incidence rates of endometrial cancer (EC) range between 15 and 25 per 100,000 women in western countries [1,2]. Commonly, a combination of clinical and histopathologic criteria, as histology, grade, lymph vascular invasion, and stage, are usually used to define EC prognosis. The same criteria are also used to tailor surgery and to select patients for adjuvant therapy. Molecular alterations have been integrated into the characterization of ECs since the Cancer Genome Atlas (TCGA) project identified four distinct groups based on molecular alterations [3,4,5,6,7,8,9,10,11]. The SWI/SNF (switch/sucrose non-fermenting chromatin remodeling) complex is the pattern of proteins more investigated in EC. Among the several subunits making up SWI/SNF, ARID1A seems to be the one more involved in the loss of function of the complex in EC. About 40% of both low- and high-grade endometrioid ECs harbor mutations in the *ARID1A* gene. On the contrary, *ARID1A* alterations have not been detected in serous endometrial carcinomas [6,11,12,13,14]. Mutations in the *ARID1A* gene may result in the loss of ARID1A protein expression, with consequent alterations in the SWI/SNF functions. This loss of function determines defects in the cell cycle checkpoint activation in response to DNA damage [15], deregulation of the signals involved in cell self-renewal, survival and proliferative capacity [16], and an alteration in the expression of genes regulated by nuclear hormonal receptors.

The multigene high-throughput technique, as next-generation sequencing (NGS), is increasingly widespread in tumors’ characterization. This approach has allowed not only to combine multigene analysis with high analytical sensitivity but also to integrate morphological and molecular features of the analyzed samples [17,18,19,20]. However, it should be considered that the huge amount of data obtained from an NGS analysis needs to be properly managed to determine correct and useful information, as, regarding *ARID1A*, it is crucial to determine if a detected mutation is disruptive or well-tolerated at the protein level. In fact, some alterations involving the “genomic level” of *ARID1A* may not lead to the loss of function of ARID1A protein, without affecting the SWI/SNF complex functions.

The aim of this study is to define the relevance of *ARID1A* alterations in a cohort of EC studying the possible association between DNA mutation (genomic level), RNA expression (transcriptomic level), and protein expression (proteomic level), to better define which *ARID1A* mutations may be considered for EC classification.

## 2. Materials and Methods

### 2.1. NGS

NGS analyses was performed on 50 endometrial carcinomas, as previously described [6]. Briefly, DNA was extracted from FFPE tissue, according to areas of interest marked on the control hematoxylin and eosin (H&E) stained slide by a pathologist (ADL). The *ARID1A* mutational status was evaluated using a multigene custom panel that allows for sequencing the whole coding sequence (CDS) of the *ARID1A* gene [6]. The results were analyzed using the Ion Reporter software (version 5.12, ThermoFisher Scientific, Waltham, MA, USA) and the Integrative Genomics Viewer 2.9 (IGV) tool (Available online: http://software.broadinstitute.org/software/igv/, accessed on 1 October 2021) [21]. The prediction of the significance of detected mutation was performed using the PolyPhen2 tool and Varsome database (https://varsome.com/, accessed on 1 October 2021).

### 2.2. IHC

Immunohistochemistry (IHC) was performed using the Benchmark Ultra immunostainer. ARID1A expression was evaluated using the rabbit anti-ARID1A polyclonal antibody (Atlas Antibodies AB, Sweden—1:90 dilution). 

**Figure 1:**
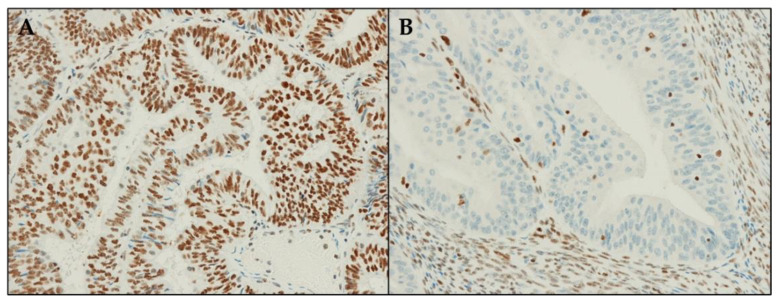
Immunohistochemical staining for ARID1A. (**A**) Preserved expression; (**B**) Loss of expression. Magnification: 200×.

Cases were independently scored using an immunoreactive scoring system by three pathologists, ADL (Pathologist 1), DS (Pathologist 2), and CC (Pathologist 3), who were blinded to the sequencing results. Abnormal ARID1A IHC was defined as a complete loss of nuclear staining in the tumor. Conversely, normal ARID1A IHC included cases with positive nuclear staining, including cases with weak staining (intensity comparable with stromal cells) and strong staining (intensity stronger than stromal cells). ARID1A nuclear staining was scored as follows: negative “loss of expression”, “positive” (weak or strong), or as “clonal loss” (i.e., the presence of a neoplastic subpopulation with loss of ARID1A immunostaining) [6,22]. In the final analysis, “clonal loss” was reclassified as “loss of expression” as this pattern corresponded to subclonal ARID1A mutations (Figure 1).

### 2.3. ARID1A RNA Expression

ARID1A expression at the transcript level was evaluated by RNAseq and by qRT-PCR in 24 cases of which 14 were *ARID1A* mutant and 10 *ARID1A* WT.

Briefly, for the RNAseq analysis, RNA quality and concentration were evaluated by the Agilent 2100 Bioanalyzer system. RNA sequencing libraries were constructed starting from 100 ng of RNA as input through the QIAseq Stranded Total RNA Lib Kit (Qiagen) following the manufacturer’s protocol. The quality and the size distribution of the libraries were checked using the High Sensitivity DNA Analysis kit (Agilent, Santa Clara, CA, USA). Subsequently, libraries were pooled and sequenced on an Illumina Nexsteq500 using the NextSeq High Output kit v2 (150 cycles) (Illumina, San Diego, CA, USA). Obtained sequences were mapped to the human genome (GRCh38) using the algorithm HISAT2 [23] and a pre-built genome index downloadable from the HISAT2 website. Then, StringTie [24] was used to assemble and quantify the transcripts in each sample. Finally, expressed transcripts were normalized using the DeSeq2 [25] package for R, low abundance features filtered, and differential gene expression analysis performed with Agilent Genespring GX software (Agilent Technologies).

ARID1A qRT-PCR was performed on cDNA synthesized from FFPE tumor samples. cDNA was obtained with the SuperScript™ IV VILO™ Master Mix kit (ThermoFisher) and ARID1A expression levels were evaluated using quantitative-PCR on the 7900HT instrument (Applied Biosystem, Waltham, MA, USA). Fold change was evaluated using the DDCt method, using GAPDH as a housekeeping gene. Primer used were: ARID1A_Fw 5′-CCAGCCGGTTCTTCGTG-3′; ARID1A_Rev 5′-ATCGGTGAAGAAGGGCGAG-3′; GAPDH_Fw 5′-CGGGAAGCTTGTCATCAAT-3′ and GAPDH_Rev 5′-GACTCCACGACGTACTCAGC-3′.

### 2.4. Structure Analysis

The ARID1A mutations were mapped on the structures available in the Protein Data Bank (PDB IDs 1RYU [26], 6LTH [27] and 6LTJ [27]) and in the PDB-Dev [28] (PDB-Dev ID: 00000056 [29]) databases. The analysis and the figure were produced by using UCSF Chimera [30].

## 3. Results

Clinicopathological characteristics of analyzed samples are reported in Table 1.

### 3.1. ARID1A Mutational Status

Twenty of 50 samples (40.0%) harbored at least one *ARID1A* mutation, for a total of 24 *ARID1A* alterations. Of these 24, 14 (58.4%) were missense mutations, 5 (20.8%) frameshift small indels, and 5 (20.8%) stop codon substitutions (Table 2, Supplementary Table S1). The mutations were found mainly in exon 20 (*n* = 8), exon 3 (*n* = 3), exon 1 (*n* = 2), exon 5 (*n* = 2), and exon 18 (*n* = 2). One mutation was detected in exons 6, 7, 8, 10, 12, 15, 19. In 3 of 22 mutated samples (13.6%), concomitant *ARID1A* substitutions were observed (Table 2, Supplementary Table S1).

According to PolyPhen2, 20 of 24 variants were scored as “damaging” for the protein function, while four were “well-tolerated”. According to the Varsome tool, 9 of 25 alterations (36.0%) were classified as “Benign/Likely benign”, 10 (40.0%) as “Pathogenic/Likely Pathogenic”, and 7 (28.0%) as “Variant of Uncertain Significance—VUS”.

Three of four mutations scored as well-tolerated by PolyPhen2 had been predicted as “Benign/Likely benign” according to the Varsome tools (Table 2, Supplementary Table S1). The other well-tolerated variant was a VUS according to Varsome (Table 2). Ten of 20 variants “damaging” according to PolyPhen2 were “Pathogenic” for Varsome, 6 were VUS, and 4 were predicted as “Benign/Likely benign” (Table 2, Supplementary Table S1).

### 3.2. RNA Expression

**Figure 2:**
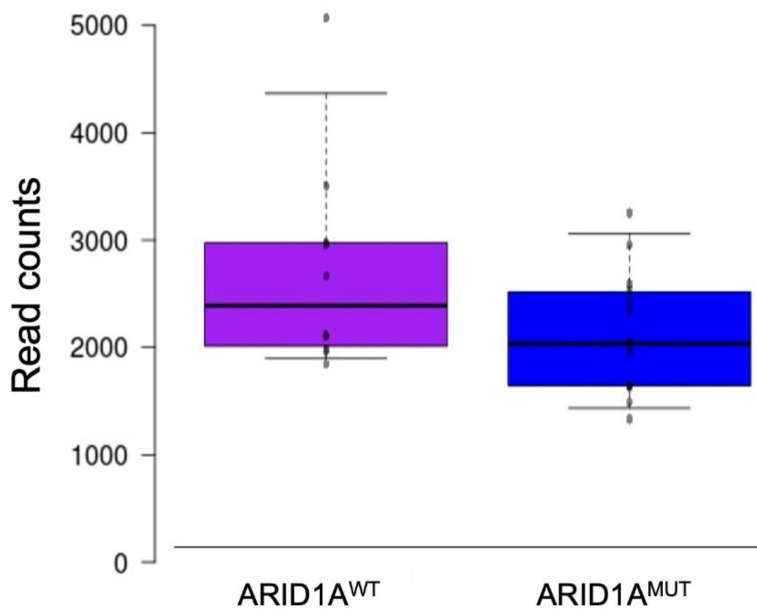
*ARID1A* expression between wildtype and mutant patients by RNAseq. ^WT^: *ARID1A* wildtype samples; ^MUT^: *ARID1A* mutant samples.

At the RNAseq level, ARID1A expression was not significantly deregulated between *ARID1A* mutant and wildtype patients (*p* = 0.08), as shown in Figure 2.

**Figure 3:**
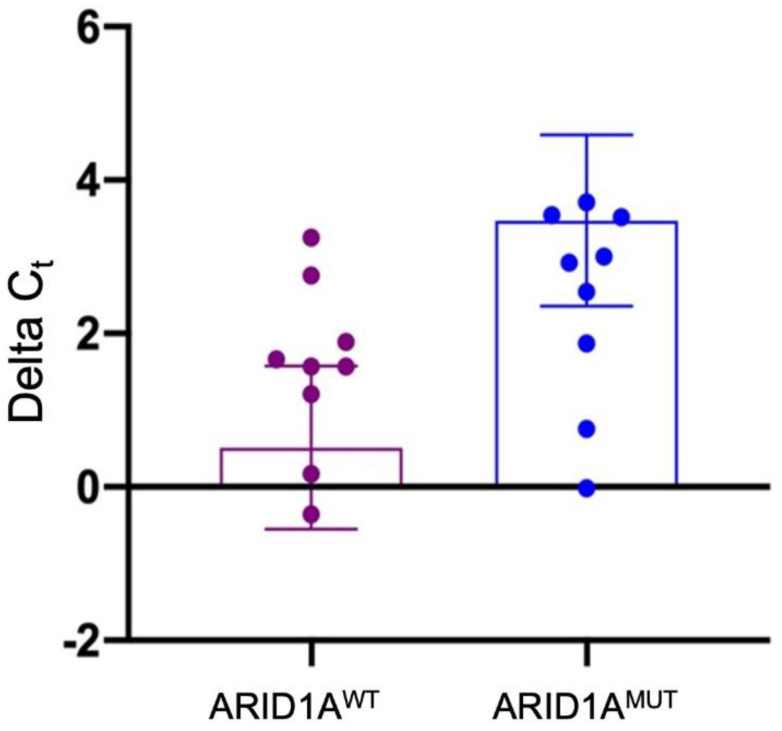
*ARID1A* expression between wildtype and mutant patients by qRT-PCR. ^WT^: *ARID1A* wildtype samples; ^MUT^: *ARID1A* mutant samples.

The same results were also confirmed at the qRT-PCR (Supplementary Table S1). Indeed, we were not able to observe a statistically significant difference between the two groups of patients (*p* = 0.06) (Figure 3).

### 3.3. ARID1A Protein Expression

ARID1A was scored as positive in 28 of 50 cases (56.0%), and negative/loss in the remaining 22 (44.0%).

Of the 28 cases with positive ARID1A staining, 24 (85.7%) did not harbor any *ARID1A* variant, while in 4 cases (14.3%) at least one mutation was detected (Table 3, Supplementary Table S1). As regarding the four “discrepant” cases: two (#1, #14) had a “benign/likely benign” *ARID1A* variants, concordant with a positive protein staining; one case (#3) harbored a disruptive mutation according to in silico tools (PolyPhen2), but with uncertain significance according to Varsome; one (#6) had two concomitant mutations, one likely benign and the other one disruptive according to in silico tools (PolyPhen2), but with an uncertain significance according to Varsome (Table 3).

Among the 22 cases with loss of ARID1A staining, in 6 cases (27.3%) no variants were detected, while in the remaining 16 (72.7%) at least one *ARID1A* mutation was observed. Of these 16 mutated cases, 10 (62.5%) harbored a frameshift/stop codon variant, while in 6 (37.5%) a missense mutation was identified (Table 4, Supplementary Table S1). Of the six cases with a missense mutation, two (#10, #17) harbored a disruptive alteration according to in silico tools (PolyPhen2), but were “benign” according to Varsome; one (#5) had a disruptive mutation according to in silico tools (PolyPhen2), but was “benign” according to Varsome; one (#16) harbored a disruptive mutation according to in silico tools (PolyPhen2), but with uncertain significance according to Varsome; one (#5) harbored two concomitant mutations, one was likely benign according to Varsome but disruptive according to in silico tools (PolyPhen2), and the other was disruptive according to in silico tools (PolyPhen2), but with an uncertain significance according to Varsome; one (#9) harbored a predicted benign variant according to PolyPhen2 and “benign” according to Varsome; the last case (#2) harbored a well-tolerated variant according to in silico tools (PolyPhen2) and was likely benign according to Varsome.

The results were defined as concordant when: (i) the PolyPhen2 tool predicted a damaging effect of the mutation on the protein, the Varsome database provided a “Pathogenic/Likely Pathogenic” result, and IHC staining was negative (protein expression loss) (10 cases, Table 5); (ii) the PolyPhen2 tool predicted a tolerated/benign effect of the mutation on the protein, the Varsome database provided a “Benign/Likely Benign” result, and IHC staining was positive (protein expression maintained) (2 cases, Table 5); (iii) no *ARID1A* mutations were detected and the IHC staining was positive (24 cases, Table 5).

A result was considered not concordant when: (i) IHC staining was lost and no *ARID1A* mutations were detectable (6 cases, Table 5); (ii) IHC staining was positive and both PolyPhen2 and Varsome predicted a “tolerated/benign” variant (1 case, Table 5).

Results were doubtful when: (i) the IHC result was concordant with only one prediction of in silico tools (i.e., only with PolyPhen2 Score OR with Varsome verdict) (2 cases, Table 5); (ii) according to Varsome, a mutation was a “variant of unknown significance—VUS” (5 cases, Table 5).

Overall, in 36 cases a perfect concordance was found between the prediction of mutation’s effect (PolyPhen2 score), integrated data about the mutation (Varsome database), and IHC staining (Table 5); in 7 cases no concordance was observed (Table 5); in 7 cases a dubious consensus was obtained (Table 5).

IHC staining was concordant with PolyPhen2 in 15 of 20 mutated cases, and in 3 cases IHC was positive in the presence of a “damaging” variant, and in 2 cases IHC was a loss, even if the *ARID1A* variant was scored as “well-tolerated” (Table 5). IHC was concordant with the Varsome verdict in 14 of 20 mutated cases; in two cases IHC staining was lost and the Varsome verdict was “VUS”, in two cases IHC staining was positive and the two concomitant ARID1A mutations were classified as “Likely Benign” and “VUS” according to Varsome, in one case IHC was loss and the Varsome verdict was “Likely Benign”, in one case IHC was positive and the Varsome verdict was “VUS” (Table 5).

### 3.4. ARID1A Structure Data

Some fragments of ARID1A have been solved in three structures deposited in the Protein Data Bank. In particular, residues 1000-1119 are present in PDB ID 1RYU, while in PDB IDs 6LTH and 6LTJ the structure for residues 1639-1746, 1802-1862, 1954-2025, 2047-2210, 2225-2285 has been solved. Moreover, in PDB IDs 6LTH and 6LTJ other fragments of the SWI/SNF complex are present, and it is possible to observe that ARID1A acts as a sort of “hub” in the C-terminal region (i.e., from residue 1639). Recently, a new ARID1A structure in the SWI/SNF complex was deposited in the PDB-Dev database (PDB-Dev ID 00000056). In the latter structure, the solved residues were 1663-1740, 1771-1783, 1804-1840, 1904-1941, 1961-2026, and 2049-2285. In other words, no structural information is available for the ARID1A N-terminal region before residue 1000 and for a large portion, comprised between residues 1119 and 1639. Even in the solved regions, there are several parts for which no structure is available. The length of the missing regions and the absence of suitable templates impede the reconstruction through homology modeling and for this reason, only the mutations that can be mapped on the available structures are discussed hereafter.

Regarding the mutations, it is possible to suppose that all the “stop” mutations (p.Arg693Ter, p.Arg1722Ter, and p.Arg1989Ter) do not allow the translation of the C-terminal region, and thus the interaction with the other proteins of the SWI/SNF complex is not possible, preventing the correct assembly of the complex itself. The same can be inferred for the “frameshift” mutations (p.Ser530fs, p.Pro728fs, p.Lys996fs, p.Gln1519fs, and p.Ser2262fs). From the frameshift mutation onwards, the protein may not fold correctly unless a second frameshift later in the sequence allows it to fold correctly again.

**Figure 4:**
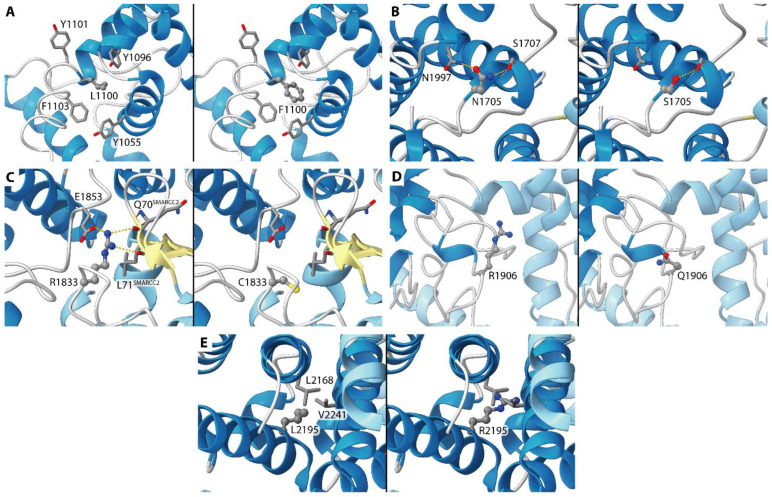
Details of ARID1A Leu1100 (**A**), Asn1705 (**B**), Arg1833 (**C**), Arg1906 (**D**), and Leu2195 (**E**) and of the neighboring residues in the available structures. In each panel, the wildtype structure and the mutant calculated in silico have been reported on the left and on the right, respectively. ARID1A helices are in blue, while helices and strands from other SWI/SNF subunits are in light blue and in light yellow. The considered residue is in ball-and-stick representation while the residues in the vicinity are in sticks. The atoms are colored according to the atom type. The hydrogen bonds have been highlighted by using yellow dashed lines.

For the missense mutations, the following can be observed from the available structures (Figure 4, the structure on which the mutation was studied is reported between parenthesis):

- p.Leu2195Arg (6LTH/6LTJ) (Case #5). This residue is located at the buried edge of a partially buried α-helix. Leu2195 forms hydrophobic contacts with ARID1A Val2168 and Leu2241. The observed mutation can cause mild to severe effects on the latter interactions considering the positive charge and the H-bond donor capability of the arginine side chain tail, even if it is difficult to predict their extent.
- p.Leu1100Phe (1RYU) (Case #12). Leu1100 is located in the ARID1A hydrophobic core and is partially accessible to the solvent. The vicinity of several ARID1A aromatic residues (Tyr 1055, Tyr1096, Tyr1101, Phe1103) can cause the formation of new interactions when Leu1100 is mutated in phenylalanine. On the other hand, it is difficult to predict the effect of this mutation on the ARID1A folding.
- p.Asn1705Ser (6LTH/6LTJ) (Case #14). This residue is located on a solvent-exposed α-helix. Asn1705 is H-bonded to ARID1A Ser1707 and Asn1997. Considering the similar length and the similar H-bond propensity of asparagine and serine, this mutation is not supposed to cause large structural effects.
- p.Arg1833Cys (6LTH/6LTJ) (Case #16). This residue is located on a solvent-exposed loop. The side chain of Arg1833 is H-bonded to Gln70 and Leu71 backbone oxygen atoms of the SMARCC2 subunit and to ARID1A Glu1853. The observed mutation can damage these interactions considering the different lengths in the side chain of an arginine residue if compared with the length of a cysteine. Moreover, cysteine is not a good H-bond donor as an arginine.
- p.Arg1906Gln (00000056) (Case #17). The mutation is on a residue that is exposed to the solvent and that does not interact with other residues from ARID1A or from other SWI/SNF proteins. A polar residue is replaced by a similar equally polar residue. Apparently, this mutation is not supposed to cause any effect.

## 4. Discussion

The integration of molecular data with histopathological features in EC is becoming more and more important since TCGA proposed to stratify EC in different groups, according to their molecular alterations (*POLE*-ultramutated; copy-number low/NSMP and hypermutated/MMRd groups; copy-number high/p53 mutant group) [31]. Several studies have investigated the importance of biomarkers in distinguishing different EC subclasses with different outcomes [3,4,5,6,7,8]. Among molecular markers relevant in EC, *ARID1A* alterations seem to play an important role in defining the clinical and prognostic features. Sequencing of the entire coding region of *ARID1A* may lead to detecting “novel” mutations and/or mutations with unknown significance regarding the effect on the protein functions [6]. To the best of our knowledge, for the first time, this study defines the relevance of *ARID1A* alterations in a cohort of ECs evaluating the possible association between DNA mutation (genomic level), RNA expression (transcriptomic level), and protein expression (proteomic level). The detection of a variant that does not influence the role of ARID1A protein should not lead to the use of this data for the molecular algorithm for classifying ECs. In our study, we have observed that *ARID1A* mutations do not influence in a statistically significant way the *ARID1A* expression level in ECs. Several in silico tools are nowadays available for predicting the possible role of gene mutations (e.g., PolyPhen2, Varsome). We have tried to classify *ARID1A* mutations identified in our cohort of ECs both using two in silico tools (PolyPhen2 and Varsome) and comparing genetic results with ARID1A protein immunostaining. In the vast majority (72%) there was a good concordance between the different methods used for classifying the detected mutations. The regions covered by the used panel allow analyzing the whole coding sequence (CDS) and 20 nucleotides spanning each exon of the *ARID1A* gene (including possible splicing sites). Eventual alterations in splicing sites will be then detectable using this panel. It should be considered that some mutations may fall into intronic regions not covered by our panel, but usually, these types of alterations do not affect RNA and protein expression. 

The p.Arg1989Ter (c.5965C>T) mutation was the most common substitution found in our cohort. This non-sense mutation is classified as pathogenic according to ClinVar (https://www.ncbi.nlm.nih.gov/clinvar/, accessed on 31 January 2022) and the Varsome database (https://varsome.com/, accessed on 31 January 2022). This mutation has been frequently described in endometrial cancer (41 entries in Cosmic database—https://cancer.sanger.ac.uk/cosmic, accessed on 31 January 2022), large intestine tumors (14 samples), pancreatic ductal adenocarcinomas (5 samples), breast carcinomas (4 entries), and gastric cancer (3 cases). This mutation has been reported also in one case of cervical squamous cell carcinoma in the TCGA pan-cancer data (https://www.cbioportal.org/, accessed on 31 January 2022). According to data reported on cBioPortal, the *ARID1A* p.Arg1989Ter mutation is likely oncogenic and will likely induce a loss of ARID1A expression, which may be related to sensitivity to EZH2 and BET inhibitors [32,33].

The presence of concomitant mutations in the *ARID1A* gene in the same sample may be explained with two different biological conditions: (i) the same clonal neoplastic cells that harbor one *ARID1A* mutation on one allele and the other alteration on the other allele; or (ii) tumor heterogeneity, i.e., different clones of neoplastic cells that harbor different *ARID1A* mutations.

For some variants, the in silico tools were not concordant, or the significance was unclear. In those cases, it is fundamental to test protein expression using IHC. Intriguingly, in six cases ARID1A was lost at IHC, even if no alterations were detectable in the *ARID1A* sequence. In these samples it is possible that a loss of protein immunostaining is due to epigenetic mechanisms [34,35], including, but not limited to, *ARID1A* copy-number aberration [36], or the microRNAs effect [37]. In five cases a mutation in the *ARID1A* sequence did not correspond to a loss of protein expression. This discrepancy may be due to missense mutations that did not affect the ARID1A protein expression, and then these cases should be considered as “not *ARID1A* mutated”. However, it should be considered that acetylation-level changes in specific histone sites in *ARID1A* mutated samples [10]; some mutations may not affect ARID1A immunostaining but could alter ARID1A function if these alterations affect acetylation sites, and then these cases should be considered as “*ARID1A* mutated samples”. Using Protein Data Bank sequences, we have also tried to further investigate if detected mutations may alter the ARID1A protein structure, but only in a few cases do we find the corresponding structures deposited. In those tumors harboring *ARID1A* mutations but with ARID1A positive immunostaining, it would be very interesting to deeply investigate if these variants may influence the assembling of ARID1A with other subunits of the SWI/SNF complex (e.g., SMARCA2/SMARCA4). In fact, the ARID1A subunit is crucial for the SWI/SNF-mediated chromatin remodeling and for mediating ARID1A-associated SWI/SNF family DNA binding. It has been previously demonstrated that mutations in *ARID1A* are associated with a worse prognosis, mainly in the No Specific Molecular Profile (NSMP) group [6]. As regarding possible therapy associated with ARID1A alterations, multiple therapeutic targets (e.g., PARP, EZH2, PIK3CA) have been extensively studied according to the mutational status of *ARID1A* [38]. The ARID1A loss has been associated with a higher sensitivity to elesclomol (an HSP-90 inhibitor with pro-apoptotic activity), in endometrial cancer cell lines [39]. Moreover, laboratory data suggest that cancer cells with ARID1A loss may be sensitive to EZH2 and BET inhibitors [32,33]. The combination of a small-molecule inhibitor of the PI3K/AKT pathway (Temsirolimus) with trabectedin (an antineoplastic agent) has shown efficacy in a cohort of patients with ovarian clear cell carcinomas harboring the *ARID1A* mutation [40,41,42].

## 5. Conclusions

In conclusion, our data demonstrated that the molecular characterization of ARID1A should be associated with IHC analysis, mainly in those cases with “novel” *ARID1A* mutations or with “uncertain” pathogenic significance. Unfortunately, due to the small number of the series and the low number of recurrences, it was not possible to evaluate the prognostic significance of ARID1A at the mutational, RNA, and protein levels. Further information about the possible effect of the mutation on ARID1A functions should be also investigated, using for example, in silico structure analysis. Moreover, using both *ARID1A* sequencing and IHC may help to also detect those cases in which loss of ARID1A functions is due to different mechanisms than DNA alterations.

## Figures and Tables

**Table 1:** Clinicopathologic characteristics of the study sample. Values are counts (percentages) or mean ± standard deviation (interquartile range).

Clinicopathologic Characteristics	*n* = 50 (%)
Age, years	63 ± 11
	(34–80)
Body mass index, kg/m^2^	27.5 ± 6.6
	(22.8–30.1)
Tumor type	
Endometrioid	38 (76.0)
Dedifferentiated/Undifferentiated	4 (8.0)
Serous	7 (14.0)
Clear cell	1 (2.0)
Grade	
1	13 (26.0)
2	15 (30.0)
3	22 (44.0)
Depth of invasion	
<50%	43 (86.0)
≥50%	7 (14.0)
Lymphovascular space invasion (LVSI)	
Absent/Focal	40 (80.0)
Diffuse	10 (20.0)
Lymph node status	
Negative	44 (88.0)
Positive	6 (12.0)
FIGO stage	
IA	33 (66.0)
IB	4 (8.0)
II	1 (2.0)
III	12 (24.0)
ARID1A Alteration	20 (40.0)
Endometrioid	17 (34.0)
Dedifferentiated/Undifferentiated	2 (4.0)
Serous	1 (2.0)
Clear cell	0 (0.0)

**Table 2:** *ARID1A* alterations observed in the analyzed cohort of ECs.

Case	ARID1A Protein Mutation	Exon	PolyPhen2 Score	Varsome Verdict
1	p.Asn209Ser	1	0.049	Likely Benign
2	p.Ala226Asp	1	0.037	Likely Benign
3	p.Gly455Glu	3	0.998	VUS
4	p.Ser530fs	3	1.000	Pathogenic
5	p.Arg596His	3	0.998	Likely Benign
	p.Leu2195Arg	20	1.000	VUS
6	p.Arg693Gln	5	0.999	VUS
	p.Ala1272Val	15	0.913	Likely Benign
7	p.Arg693Ter	5	1.000	Pathogenic
8	p.Pro728fs	6	1.000	Pathogenic
9	p.Gly768Asp	7	0.181	VUS
10	p.Ala900Thr	8	0.984	Benign
11	p.Lys996fs	10	1.000	Pathogenic
12	p.Leu1100Phe	12	1.000	VUS
	p.Arg1446Gln	18	0.999	VUS
	p.Arg1989Ter	20	1.000	Pathogenic
13	p.Gln1519fs	18	1.000	Pathogenic
14	p.Asn1705Ser	19	0.137	Benign
15	p.Arg1722Ter	20	1.000	Pathogenic
16	p.Arg1833Cys	20	0.999	VUS
17	p.Arg1906Gln	20	0.996	Benign
18	p.Arg1989Ter	20	1.000	Pathogenic
19	p.Arg1989Ter	20	1.000	Pathogenic
20	p.Ser2262fs	20	1.000	Pathogenic

VUS: Variant of uncertain significance.

**Table 3:** Cases with positive ARID1A protein staining but harboring *ARID1A* DNA mutation.

Case	Aminoacidic Change	PolyPhen2 Score	Varsome Verdict	IHC
1	p.Asn209Ser	0.049	Likely Benign	Positive
3	p.Gly455Glu	0.998	VUS	Positive
6	p.Arg693Gln p.Ala1272Val	0.999 0.913	VUS Likely Benign	Positive
14	p.Asn1705Ser	0.137	Benign	Positive

IHC: immunohistochemistry; VUS: variant of uncertain significance.

**Table 4:** Cases with loss IHC staining and missense *ARID1A* mutation.

Case	Aminoacidic Change	PolyPhen2 Score	Varsome Verdict	IHC
2	p.Ala226Asp	0.037	Likely Benign	Loss
5	p.Arg596His p.Leu2195Arg	0.998 1.000	Likely Benign VUS	Loss
9	p.Gly768Asp	0.181	VUS	Loss
10	p.Ala900Thr	0.984	Benign	Loss
16	p.Arg1833Cys	0.999	VUS	Loss
17	p.Arg1906Gln	0.996	Benign	Loss

IHC: immunohistochemistry; VUS: variant of uncertain significance.

**Table 5:** Comparison between ARID1A mutational status, in silico prediction effect, and ARID1A protein expression. WT: Wild-Type; IHC: immunohistochemistry; VUS: Variant of uncertain significance; ?: dubious consensus.

#	*ARID1A* Mutational Status	POLYPHEN2 Score	Varsome Verdict	IHC Staining	Consensus
24 cases	WT	/	/	Positive	OK
6 cases	WT	/	/	Loss	NO
1	p.Asn209Ser	0.049	Likely Benign	Positive	OK
2	p.Ala226Asp	0.037	Likely Benign	Loss	NO
3	p.Gly455Glu	0.998	VUS	Positive	?
4	p.Ser530fs	1.000	Pathogenic	Loss	OK
5	p.Arg596His	0.998	Likely Benign	Positive	?
	p.Leu2195Arg	1.000	VUS		
6	p.Arg693Gln	0.999	VUS	Positive	?
	p.Ala1272Val	0.913	Likely Benign		
7	p.Arg693Ter	1.000	Pathogenic	Loss	OK
8	p.Pro728fs	1.000	Pathogenic	Loss	OK
9	p.Gly768Asp	0.181	VUS	Loss	?
10	p.Ala900Thr	0.984	Benign	Loss	?
11	p.Lys996fs	1.000	Pathogenic	Loss	OK
12	p.Leu1100Phe	1.000	VUS	Loss	OK
	p.Arg1446Gln	0.999	VUS		
	p.Arg1989Ter	1.000	Pathogenic		
13	p.Gln1519fs	1.000	Pathogenic	Loss	OK
14	p.Asn1705Ser	0.137	Benign	Positive	OK
15	p.Arg1722Ter	1.000	Pathogenic	Loss	OK
16	p.Arg1833Cys	0.999	VUS	Loss	?
17	p.Arg1906Gln	0.996	Benign	Loss	?
18	p.Arg1989Ter	1.000	Pathogenic	Loss	OK
19	p.Arg1989Ter	1.000	Pathogenic	Loss	OK
20	p.Ser2262fs	1.000	Pathogenic	Loss	OK

## Data Availability

The data about RNAseq are available upon request. All other data presented in this study are available in the article.

## Supplementary Materials

The following are available online at https://www.mdpi.com/article/10.3390/diagnostics12030592/s1, Table S1: Sequencing, RNA expression, and IHC results of the 50 analyzed cases.
