# Supplementary material for: Relevance of ARID1A Mutations in Endometrial Carcinomas
Source: Diagnostics (Basel). 2022 Feb 25;12(3):592. doi: 10.3390/diagnostics12030592 (PMC8947028; doi:10.3390/diagnostics12030592)
Supplement: Supplementary file 1 [file diagnostics-12-00592-s001.zip › diagnostics-1607727-supplementary.pdf]

Table S1: Sequencing, RNA expression, and IHC results of the 50 analyzed cases.

#	Histological Diagnosis	ARID1A status	ARID1A Mutation	Ex	IHC	RNA Level (Delta Ct)
1	S	Mut	p.Asn209Ser	1	Positive	1.21
2	DED	Mut	p.Ala226Asp	1	Loss	
3	E	Mut	p.Gly455Glu	3	Positive	1.87
4	E	Mut	p.Ser530fs	3	Loss	2.92
5	DED	Mut	p.Arg596His<br>p.Leu2195Arg	3<br>20	Positive	
6	E	Mut	p.Arg693Gln<br>p.Ala1272Val	5<br>15	Positive	1.82
7	E	Mut	p.Arg693Ter	5	Loss	3.01
8	E	Mut	p.Pro728fs	6	Loss	
9	E	Mut	p.Gly768Asp	7	Loss	
10	E	Mut	p.Ala900Thr	8	Loss	
11	E	Mut	p.Lys996fs	10	Loss	
12	E	Mut	p.Leu1100Phe<br>p.Arg1446Gln<br>p.Arg1989Ter	12<br>18<br>20	Loss	3.79
13	E	Mut	p.Gln1519fs	18	Loss	3.54
14	E	Mut	p.Asn1705Ser	19	Positive	
15	E	Mut	p.Arg1722Ter	20	Loss	
16	E	Mut	p.Arg1833Cys	20	Loss	
17	E	Mut	p.Arg1906Gln	20	Loss	
18	E	Mut	p.Arg1989Ter	20	Loss	
19	E	Mut	p.Arg1989Ter	20	Loss	0.17
20	E	Mut	p.Ser2262fs	20	Loss	-8.59
21	S	WT	/	/	Positive	2.76
22	E	WT	/	/	Positive	-0.36
23	E	WT	/	/	Loss	
24	E	WT	/	/	Positive	12.87
25	E	WT	/	/	Positive	
26	DED	WT	/	/	Positive	1.57
27	E	WT	/	/	Positive	

28	E	WT	/	/	Positive	1.57
29	E	WT	/	/	Positive	
30	E	WT	/	/	Positive	
31	E	WT	/	/	Positive	
32	E	WT	/	/	Loss	
33	E	WT	/	/	Positive	
34	DED	WT	/	/	Positive	1.89
35	E	WT	/	/	Positive	
36	E	WT	/	/	Positive	1.66
37	S	WT	/	/	Positive	
38	E	WT	/	/	Loss	
39	S	WT	/	/	Positive	
40	S	WT	/	/	Positive	2.54
41	E	WT	/	/	Positive	
42	S	WT	/	/	Positive	
43	E	Wt	/	/	Positive	3.25
44	E	WT	/	/	Loss	
45	E	WT	/	/	Positive	0.76
46	E	WT	/	/	Loss	
47	E	WT	/	/	Loss	
48	S	WT	/	/	Positive	
49	CCC	WT	/	/	Positive	
50	E	WT	/	/	Positive	3.52

S: Serous endometrial carcinoma

E: Endometrioid carcinoma
CCC: Clear Cell endometrial Carcinoma
DED: dedifferentiated/undifferentiated carcinoma
Mut: ARID1A mutated
WT: ARID1A wild-type
Ex: Exon
